# Supplementation with a Symbiotic Induced Neuroprotection and Improved Memory in Rats with Ischemic Stroke

**DOI:** 10.3390/biomedicines12010209

**Published:** 2024-01-17

**Authors:** Yolanda Cruz-Martínez, Leslie Aguilar-Ponce, Alejandra Romo-Araiza, Almudena Chávez-Guerra, Susana Martiñón, Andrea P. Ibarra-García, Stella Arias-Santiago, Vanessa Gálvez-Susano, Antonio Ibarra

**Affiliations:** 1 Centro de Investigación en Ciencias de la Salud (CICSA), Facultad de Ciencias de la Salud, Universidad Anáhuac México Campus Norte, Huixquilucan CP 52786, Edo. de México, Mexico; yolanda.cruz@anahuac.mx (Y.C.-M.); iantonio65@yahoo.com (L.A.-P.); alejandra.romo@anahuac.mx (A.R.-A.); ana.chavezgu@anahuac.mx (A.C.-G.); silvia.martinon@anahuac.mx (S.M.); andrea_ibarra@anahuac.mx (A.P.I.-G.); stella.arias@anahuac.mx (S.A.-S.); jvgs_yaem@hotmail.com (V.G.-S.); 2 Laboratorio de Inmunología en Adicciones, Subdirección de Investigaciones Clínicas, Instituto Nacional de Psiquiatría Ramón de la Fuente Muñiz, Tlalpan CP 14050, Ciudad de México, Mexico

**Keywords:** cognitive impairment, symbiotic, cerebral ischemia, BDNF, TNF-α, tMCAO, *Enterococcus faecium*, cytokines, neuroprotection, memory

## Abstract

After an ischemic stroke, various harmful mechanisms contribute to tissue damage, including the inflammatory response. The increase in pro-inflammatory cytokines has been related to greater damage to the neural tissue and the promotion of neurological alterations, including cognitive impairment. Recent research has shown that the use of prebiotics and/or probiotics counteracts inflammation and improves cognitive function through the production of growth factors, such as brain-derived neurotrophic factor (BDNF), by reducing inflammatory molecules. Therefore, in this study, the effect of the symbiotic inulin and *Enterococcus faecium* on neuroprotection and memory improvement was evaluated in a rat model of transient middle cerebral artery occlusion (tMCAO). In order to accomplish this, the animals were subjected to ischemia; the experimental group was supplemented with the symbiotic and the control group with the vehicle. The neurological deficit as well as spatial and working memory were evaluated using the Zea Longa scale, Morris water maze, and the eight-arm maze tests, respectively. Infarct size, the levels of BDNF, and tumor necrosis factor-alpha (TNF-α) were also assessed. The results show that supplementation with the symbiotic significantly diminished the neurological deficit and infarct size, improved memory and learning, increased BDNF expression, and reduced TNF-α production. These findings provide new evidence about the therapeutic use of symbiotics for ischemic stroke and open up the possibilities for the design of further studies.

## 1. Introduction

Ischemic stroke is caused by a significant reduction in the blood flow to the vessels that supply the brain, causing the activation of several mechanisms such as biochemical dysfunction, an increment of intracellular calcium, excitotoxicity, and activation of lytic enzymes that lead to cell death and, ultimately, a region of damaged neural tissue, or ischemic core [1,2]. Later on, there is a significant increase in free radicals and inflammatory mediators such as IL-6, IL-1β, and TNF-α that contribute to the progression of injured neural tissue [3]. If therapeutic intervention is not carried out, the viable tissue surrounding the infarct area, or ischemic penumbra, dies, and thus the volume of the ischemic core increases.

The cellular changes in neural tissue involve the activation of the microglia and astrocytes, triggering the production of inflammatory mediators even in chronic phases after stroke, especially in areas such as the motor cortex and hippocampus [4]. These events lead to the dysfunction of neurons, resulting in neurological deficits and impairment in memory and learning [2,4,5]. The clinical consequences of stroke will depend on the affected brain region, the intensity of the infarction, and the promptness of medical intervention [6]. The most frequent sequelae are motor alterations and cognitive impairment, mainly in memory, attention, and executive function, including decision-making, working memory, and cognitive flexibility [7]. 

Recent studies have established that dysbiosis is associated with poor recovery and prognosis after stroke since it increases neuroinflammation and accelerates the progression of cognitive impairment [8,9]. In fact, the composition of the intestinal microbiota could be crucial for the development of stroke. Dysbiosis is associated with a higher risk of stroke since it promotes low-grade inflammation and oxidative stress, contributing to vascular lesions [9,10]. In addition, it has been observed that there is a correlation between the type of pathogenic bacteria in the microbiome and the severity of the neurological dysfunctions caused by stroke [9]. Therefore, restoring the microbiota could become a therapeutic strategy. 

In order to restore the microbiome, “a mixture of living microorganisms (probiotics) and substrates (prebiotics) used selectively by microorganisms that confer a beneficial effect on the health of the host” has been proposed [11]. 

Inulin is a fructan with prebiotic characteristics. In vitro studies have shown that it has anti-inflammatory properties by inhibiting the NF-Kβ pathway [12]. It modulates the composition of microbiota and induces the production of short-chain fatty acids (SCFAs), which also have anti-inflammatory effects [13]. In addition, inulin stimulates the growth of *Enterococcus faecium* [14], a microorganism with the ability to reduce the expression of pro-inflammatory cytokines and increase the production of SCFAs, as well as neurotrophic factors [15]. 

The use of the symbiotic made with *E. faecium* and agave inulin has shown a reduction in IL-1β and TNF-α expression in aged [15] and obese rats with cognitive impairment. It also increases the expression of BDNF and enhances excitatory synaptic transmissions in the cerebral cortex and hippocampus, improving memory and cognition [16,17,18]. As a consequence of its anti-inflammatory effects, the symbiotic could also promote neuroprotection after stroke. 

In the present study, we evaluated the effect of supplementation with *E. faecium* and agave inulin on neuroprotection and cognition in a murine model of cerebral ischemia. 

## 2. Materials and Methods

### 2.1. Experimental Design

For this study, 45 male Sprague-Dawley rats, aged three and a half months and weighing an average of 330 ± 20 g, were obtained from the Bioterium of Anáhuac University. The housing conditions of the rats (temperature and humidity) were managed by using automated controlled racks, with food and water available ad libitum, in a 12 h light/dark cycle room, before and after cerebral ischemia was induced using the transient middle cerebral artery occlusion (tMCAO) method.

**Figure 1:**
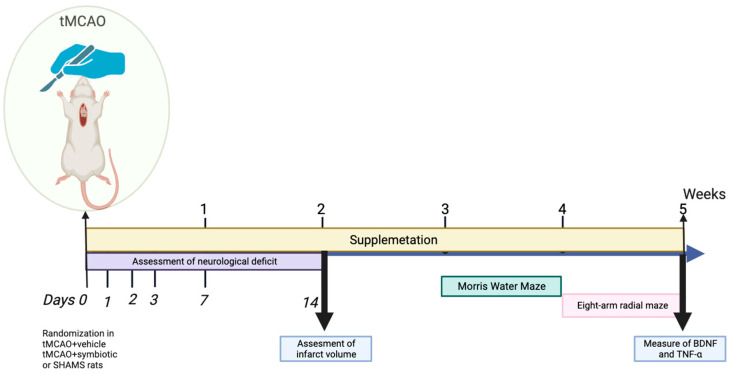
Experimental design.

To explore the effect of the symbiotic on cognitive damage in rats with stroke, we decided to perform a preliminary experiment. Rats were randomly assigned into three groups (15 rats per group) using the GraphPad QuickCals program (https://www.graphpad.com/quickcalcs/randomize1/ accessed on 8 November 2023): (1) tMCAO+ vehicle (control group), (2) tMCAO+ symbiotic (symbiotic-supplemented group), and sham-operated+ vehicle. The control and treated groups were subjected to cerebral ischemia while the sham one was only subjected to the surgical procedure without ischemia. The neurological deficit was evaluated using the Zea Longa scale [19] at 1, 2, 3, 7, and 14 days after ischemia. In order to measure the volume of cerebral infarction, 5 rats were randomly chosen from each group and then euthanized 14 days after ischemia. Three weeks after ischemia, the remaining rats (10 per group) were evaluated for spatial memory and, one week later, their working memory was assessed as well. After clinical evaluations, five weeks after tMCAO, the rats were euthanized in order to measure the hippocampal concentrations of BDNF and TNF-α. Data acquisition was performed using a double-blind design to avoid biases. The experimental design is depicted on the timeline of Figure 1.

### 2.2. Cerebral Ischemia Model

Cerebral ischemia was induced using tMCAO, a procedure that was previously described by Zea Longa in the late 1980s [19]. For this experiment, the rats were anesthetized with a facial mask and 4% isoflurane in oxygen, until a deep anesthetic state was reached; for the rest of the surgery, the concentration of isoflurane was lowered to 1.5%. The body temperature of the rats was maintained at 37 °C using a warm pad until their recovery from the anesthesia. During the procedure, five arteries were identified: the left common carotid (LCC), the internal carotid artery (ICA), the external carotid artery (ECC), and the pterygoid and occipital arteries; the last two were cauterized. A 3-0 nylon monofilament was inserted 18 mm through the ECC until the middle cerebral artery (MCA) was reached. The occlusion was left for 90 min, and afterward, the monofilament was removed, allowing the cerebral blood flow to be restored and the procedure finished with the corresponding stitches. The severity of tMCAO was evaluated by determining the regional cerebral blood flow using a laser Doppler flowmeter (Moor Instruments, Devon, UK). A reduction of around 85% in cerebral perfusion was considered to be focal ischemia. For postoperative care, the animals received acetaminophen 200 mg/kg every 12 h and enrofloxacin 10 mg/kg every 24 h, for 3 days.

### 2.3. Administration of the Vehicle and Symbiotic

The three groups were supplemented via oral probe; the control and sham groups received one milliliter of water (vehicle of treatment), and the supplemented group received a symbiotic mix of *E. faecium* and agave inulin [4 × 10^8^ CFU and 860 mg/kg, respectively [16]] mixed with the same vehicle. The administration of the vehicle or symbiotic mix was performed daily for 5 weeks. The animals chosen for infarct size evaluation were supplemented for only 14 days. 

### 2.4. Zea Longa Scale

The Zea Longa scale [19] was used to assess the neurological deficit during days 1, 2, 3, 7, and 14 after the tMCAO was performed. This scale provided a score from 0 to 4 points according to the neurological deficit achieved: 0 indicating no neurological deficit, 1 for not being able to lift the right paw, 2 for circling to the left, 3 for decaying to the right, and 4 indicating not being able to walk or referring to a decreased level of consciousness.

### 2.5. Morris Water Maze

The Morris water maze (MWM) was used to evaluate spatial and associative memory. The rats were placed in a large circular pool (120 cm of diameter) full of water (21–22 °C) and were required to escape to a hidden platform (10 cm of diameter) located 2 cm below the water surface in the south-west quadrant (SWQ) of the pool. The rats were given 3 visual clues shallowly, equidistant from each other, to help them find the location of the platform. The animals underwent a 5-day training protocol consisting of four dives per day, with a maximum duration of 60 s of swimming and 20 s of rest on the platform, for 5 consecutive days. The initial position of the rats was different every day. During the 5-day training protocol, the platform was removed, and the rats were allowed to swim for 60 s to analyze the time they spent in the SWQ versus the time they spent in the remaining quadrants. All trials were recorded using a computerized system (Smart v3.0.02 Panlab Harvard Apparatus, Barcelona, Spain) to determine the latency time taken by each rat to reach the platform below the surface from their initial position, based on the time coordinates of each rat.

### 2.6. Eight-Arm Radial Maze

An eight-arm radial maze was used to evaluate working and reference memory and provided an indicator of memory deficit. The test consisted of an eight-arm maze in which food was placed on each arm. In 4 of them, food was displayed so that it could not be observed by the rat, but was accessible, whereas in the other 4 arms, access was not provided. This was applied to prevent the animals from be guided by smell. The main objective of this test was to evaluate the working and reference memory simultaneously. The animals have to find the arms with accessible food using extra-maze clues, while avoiding the arms where they already ate the food (working memory), but also avoiding the arms where the food is not accessible to them (reference memory) [20]. The test results were reported as the percentage of correct responses obtained by dividing the number of correct entries in the arms with accessible food by the total number of entries.

### 2.7. Brain Infarct Volume

Fourteen days after ischemia, five rats (randomly chosen) from each group were euthanized with an overdose of pentobarbital. The brains were extracted without perfusing them and placed at a temperature of −20 Celsius for 15 min, with the purpose of facilitating the slicing process. Each brain was placed in a matrix and 2 mm thick coronal sections were made. The slices were placed in a Petri dish and immersed in 25 mL of 1.5% 2,3,5-triphenyl tetrazolium (TTC) solution (Sigma-Aldrich, St. Louis, MO, USA), for 15 min at 37 °C. The TTC-stained sections were fixed with 4% paraformaldehyde. Afterward, digitized photographs (Canon EOS Rebel SL2; Tokyo, Japan) were obtained. The infarct area was determined using the Image J image analyzer (Image J, National Institutes of Health, Bethesda, MD, USA). The infarct volume was calculated using the Reglodi method, considering edema (EA). The formula used was EA-infarct volume = infarct volume × (contralateral hemisphere/ipsilateral hemisphere) [21].

### 2.8. Enzyme-Linked Immunosorbent Assay (ELISA)

An ELISA test was performed to measure the levels of brain-derived neurotrophic factor (BDNF) and TNF-α cytokine in 7 rats of each group. The rats were euthanized using a lethal injection of phentobarbital and then decapitated so that the brain could be removed, and the hippocampus dissected: the brain was cut along the longitudinal fissure and the two hemispheres were separated, from both of which the cerebellum and the olfactory bulb were removed. Subsequently, with a spatula, the thalamus and the corpus callosum were removed to expose the entire hippocampus. Once identified, it was separated from the cerebral cortex to be completely removed [22]. 

The extracted tissue was frozen at a temperature of −80 °C until the study was carried out. The tissue samples were homogenized in a buffer substance at a ratio of 10:1 buffer–tissue weight; the homogenized samples were centrifuged at 14,000× *g* for half an hour and the supernatant was used to perform an ELISA test. Each sample was run in quintuplicate for each ELISA test, following the instructions provided by the manufacturers (ChemiKineTM from Merck, Darmstadt, Germany, and BioLegend, San Diego, CA, USA).

### 2.9. Statistical Analysis

Statistical analysis was performed using the Prism 5 software (Prism 5.01, GraphPad Software Inc., San Diego, CA, USA). Data are expressed as mean ± standard deviation and a significance level of *p* < 0.05 was considered. The Shapiro–Wilk normality test was used to analyze the normality of each data set. Neurological deficit was evaluated by using ANOVA for repeated measures. Two-way ANOVA for repeated measures, Student’s *t*-test, and Cohen’s d tests were used to assess spatial memory. Working memory, BDNF and TNF-α concentrations, and infarct volume were analyzed using one-way ANOVA and Student’s *t*-test. 

## 3. Results

### 3.1. Symbiotic Supplementation Reduced the Neurological Deficit Observed after tMCAO

**Figure 2:**
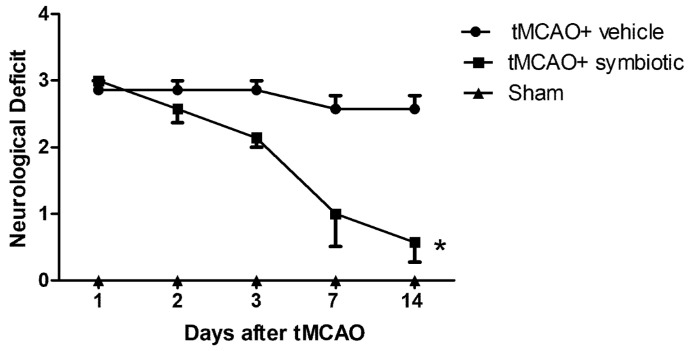
The supplementation with the symbiotic *E. faecium* and agave inulin enhances neurological recovery in rats with tMCAO. Evaluations were performed on days 1, 2, 3, 7, and 14 post-tMCAO. Each point represents mean ± SD of 15 rats per group; * *p* < 0.0001, ANOVA for repeated measures.

After the rats were subjected to tMCAO, neurological deficit was assessed at 1, 3, 7, and 14 days post-ischemia. The evaluations of the neurological deficit showed that on the first day after ischemia, the control group and the one supplemented with the symbiotic presented a similar neurological deficit (control: 2.8 ± 0.3; symbiotic-supplemented: 3.0 ± 0.02; mean ± SD; *p* > 0.05, Student’s *t*-test, Figure 2). However, at the end of this evaluation (14 days after ischemia), there was a significant improvement in the symbiotic-supplemented group. The neurological deficit was significantly reduced in these animals, (0.57 ± 0.2) in comparison with the control rats (2.57 ± 0.2; *p* < 0.0001, ANOVA for repeated measures). The sham rats did no present any deficit.

### 3.2. Symbiotic Supplementation Reduced the Infarct Volume after tMCAO

**Figure 3:**
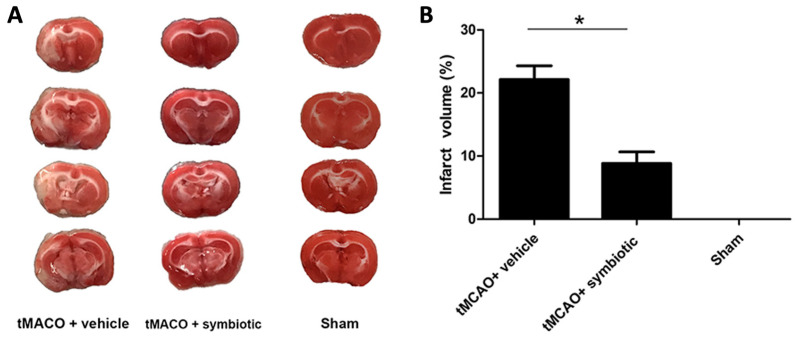
The supplementation with the symbiotic *E. faecium* and agave inulin reduces infarct volume in rats with tMCAO. (**A**) Representative TTC staining of cerebral infarction in comparable coronal sections in rats treated with the symbiotic, vehicle, and sham at 14 days post-tMCAO. (**B**) Quantification of infarct volume based on TTC staining at 14 days post-tMCAO in rats supplemented with the symbiotic, control group, or sham-operated rats. n = 5 mean ± SD (* *p* = 0.003). Student’s *t*-test was employed.

Fourteen days after tMCAO or the sham operation, five rats from each group (randomly chosen) were euthanized for morphological assessment. The 2,3,5-triphenyltetrazolium chloride (TTC) staining method was used to determine the volume of cerebral infarction. Figure 3 shows that the symbiotic-supplemented rats presented a significant reduction in infarct volume (8.84 ± 1.8; mean ± SD) as compared to the control animals (22.15 ± 2.2; *p* = 0.003, Student´s *t*-test). Animals subjected only to the surgical procedure (sham-operated) did not present infarct signals.

### 3.3. Supplementation with the Symbiotic Enhances Spatial Memory after tMCAO

**Figure 4:**
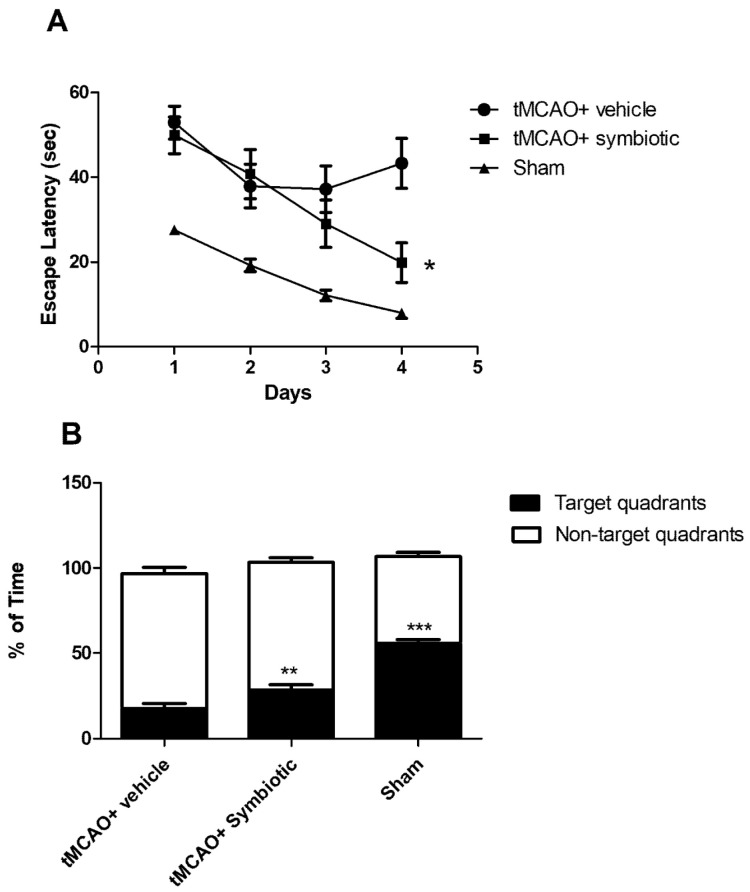
Evaluation of spatial and associative memory in rats with tMCAO supplemented with symbiotic *E. faecium* and agave inulin. (**A**) Escape latency time (seconds) to the platform hidden in the south-west quadrant (SWQ). (**B**) Time spent (percentage) in the target quadrant compared to the time spent in the non-target quadrants. n = 10 mean ± SD; * *p* = 0.02; two-way ANOVA for repeated measures followed by post hoc Bonferroni test; ** *p* = 0.01 vs. control, *** *p* < 0.001 vs. symbiotic. Student’s *t*-test.

In order to evaluate the effect of the symbiotic *E. faecium* and agave inulin on spatial memory three weeks after ischemia, the remaining rats (ten rats per group) were assessed with the Morris water maze test. As expected, the rats subjected to tMCAO showed a significant increase in scape latency time as compared to the sham-operated ones (Figure 4A). However, the time of symbiotic-supplemented rats was lower than that presented by control animals. The interaction between therapy and time was also statistically significant when comparing the symbiotic-supplemented to the control rats (F = 3.37; *p* = 0.02; ANOVA for repeated measures followed by post hoc Bonferroni test). The last day of acquisition showed a significant difference between the tMCAO-studied groups (symbiotic-supplemented: 19.85 ± 4.66, mean ± SD; control: 43.28 ± 5.89; *p* = 0.01 Student´s *t*-test). In order to know the size of the effect between the groups subjected to tMCAO, we also calculated Cohen’s d [23], obtaining a value of 1.14 (large effect) in spatial memory. 

Memory retention was also analyzed on the sixth day by removing the platform and comparing the time spent in the target quadrant to the average time spent in the non-target quadrants (Figure 4B). The sham-operated group spent the longest time in the target quadrant (55.7 ± 2.29). In the case of the tMCAO-subjected rats, the group supplemented with the symbiotic spent more time (28.36 ± 3.14, mean ± SD) when compared to the control one (17.69 ± 2.87; *p* = 0.01, Student´s *t*-test, Figure 4B).

### 3.4. Work Memory Was Also Enhanced by Symbiotic Supplementation

**Figure 5:**
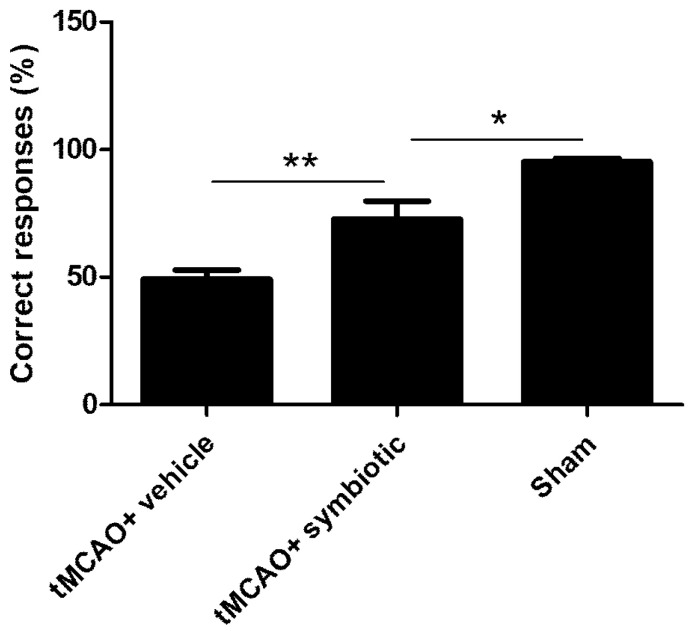
Evaluation of working memory in rats with tMCAO supplemented with symbiotic *E. faecium* and agave inulin. Percentage of correct entries to the eight-armed radial maze. n = 10 mean ± SD; * *p* < 0.05, ** *p* = 0.003, Student’s *t*-test.

With the aim of evaluating the effect of the symbiotic in other domains of memory, the rats were assessed for working memory using an eight-arm radial maze one week after spatial memory evaluation. In this task, working memory was assessed when the rats entered each arm once. In this case, the sham-operated rats performed again the best for this task (95.13 ± 1.44 mean ± SD; Figure 5). In the case of the rats subjected to tMCAO, the symbiotic-supplemented animals showed better performance compared to the control animals (Figure 5). The percentage of correct responses in the symbiotic-supplemented group was significantly higher (72.74 ± 7.02) than that observed in the control one (49.08 ± 3.71; *p* = 0.003, Student´s *t*-test).

### 3.5. Hippocampus of Symbiotic-Supplemented Rats Presented an Increase in BDNF and a Reduction in TNF-α

**Figure 6:**
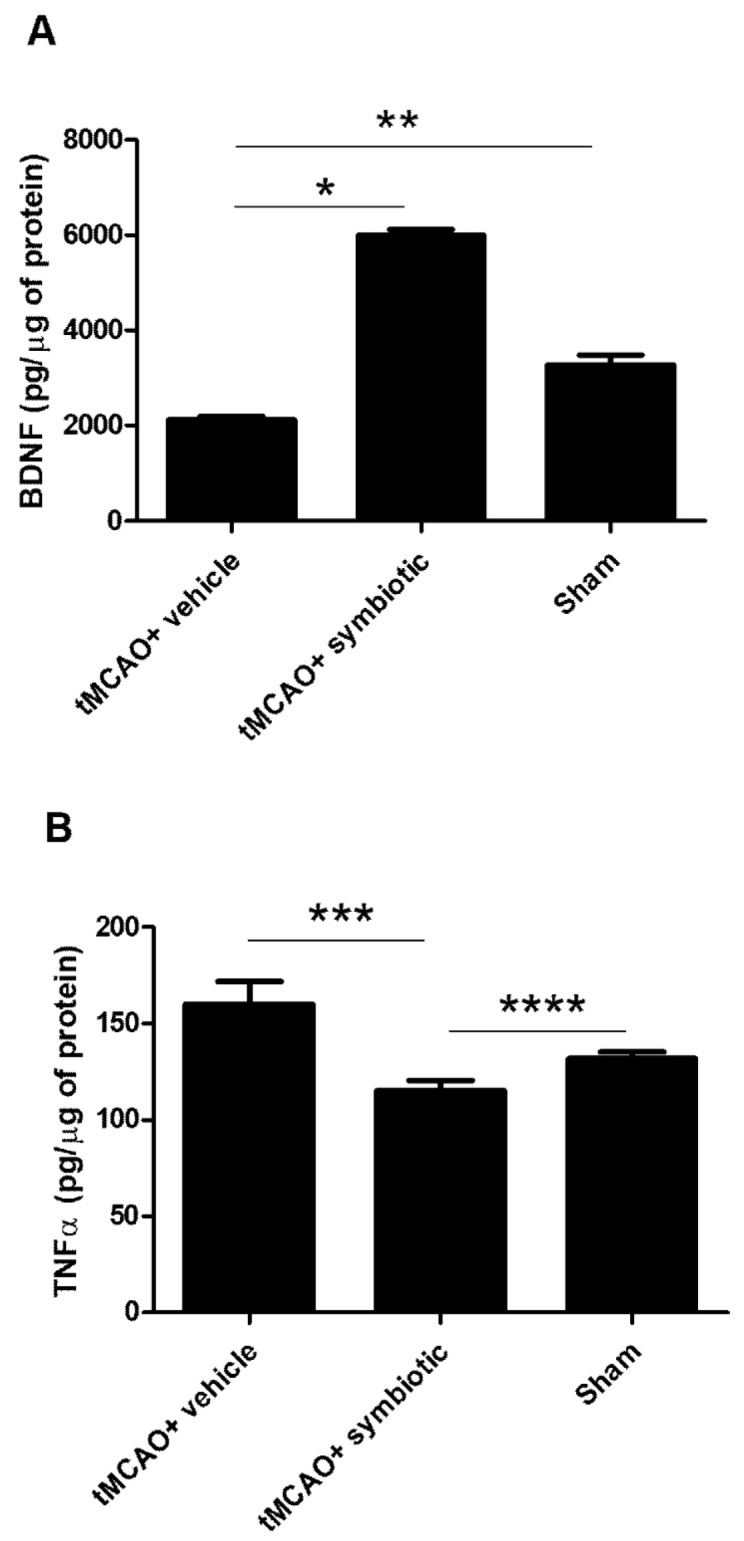
BDNF and TNF-α concentration in rats with tMCAO supplemented with the symbiotic *E. faecium* and agave inulin. (**A**) Concentrations of BDNF. (**B**) Concentrations of TNF-α. n = 7 mean ± SD (* *p* ≤ 0.0001); ** *p* < 0.05, *** *p* = 0.01, **** *p* = 0.05. One-way ANOVA followed by Tukey´s post hoc test.

As the hippocampus is strongly related to memory establishment and this cognitive function depends on BDNF availability [16], we decided to analyze the concentrations of BDNF in this brain region. As shown in Figure 6A, symbiotic supplementation induced an increase in BDNF levels. The concentrations of this molecule were significantly higher in the symbiotic-supplemented rats (5994 ± 124.2; pg/µg of protein, mean ± SD) than those observed in the control (2122 ± 71.94; *p* < 0.0001) and sham-operated animals (3279 ± 202.7; *p* < 0.05, one-way ANOVA followed by Tukey´s post hoc test). On the other hand, inflammation is one of the phenomena inducing cognitive impairment but also participating in tissue damage after ischemia, especially in the hippocampus. We then evaluated the levels of TNF-α, one of the main cytokines in the inflammatory response observed after stroke (Figure 6B). The concentration of this cytokine was significantly reduced in the symbiotic-supplemented rats (115.2 ± 5.3; pg/µg of protein, mean ± SD) when compared to those observed in the control and sham-operated animals (160.1 ± 11.8; *p* = 0.01 and 132.0 ± 3.3; *p* = 0.05, respectively; one-way ANOVA followed by Tukey´s post hoc test). 

## 4. Discussion

Recent investigations have proposed the use of symbiotics as therapeutic alternatives for stroke [24,25]. The results of the present investigation show that supplementation with the symbiotic *Enterococcus faecium* and agave inulin as early as 14 days post-stroke improves neurological recovery in a tMCAO model. These results provide significant evidence on the neuroprotective effect that symbiotic supplementation can exert by limiting the size of the infarct, which in turn improves neurological recovery. 

In addition, we observed a decrease in TNF-α and an increase in BDNF concentrations in the hippocampus after supplementation with *Enterococcus faecium* and agave inulin. TNF-α is a key cytokine for inducing neuroinflammation [26]. It has been observed that this cytokine contributes to the exacerbation of tissue injury after stroke [27] through different mechanisms, such as mitochondrial dysfunction, leading to apoptosis [28]. It also mediates endothelial necroptosis by increasing the permeability of the blood –brain barrier (BBB) [29] and reducing neurogenesis [30] and neuroplasticity after the ischemic event [31]. On the other hand, BDNF is a growth factor that stimulates cell survival and neural differentiation. The increase in BDNF may mediate the survival of injured neurons through the activation of the PI3K/Akt pathway and MAPK/ERK, inhibiting apoptosis [32]. This molecule also promotes neurotransmission and synaptic plasticity [33]; its deficiency is related to cognitive impairment after an ischemic event [34] and contributes to the prognosis of the neurological outcome. 

The changes in the expression of BDNF and TNF-α could be responsible, at least in part, for the neuroprotective effect observed in the supplemented rats. These changes are possible as the symbiotic can selectively increase beneficial bacteria in the intestine as well as levels of short-chain fatty acids [35], such as butyrate, which in turn increases BDNF expression by inhibiting histone deacetylation [12]. Butyrate is obtained as a fermentative product of inulin by *E. faecium* [15]. In addition, *Enterococcus faecium* and agave inulin individually have been shown to possess anti-inflammatory characteristics [11,14]. It has been proven that inulin itself has the ability to reduce the IFN-gamma production by the CD4+ T cells and to increase T reg cells, which express high amounts of IL-10 and a low quantity of IL-6 [36]. Furthermore, inulin also suppresses M1 macrophages and polarizes the M2 phenotype [12], increasing the peripheral blood monocytic myeloid-derived suppressor cells [37]. Moreover, *E. faecium* reduces the IL-8, TNF-α, and IL-6 levels in the macrophages [38], and free radicals overall, and increases the IL-10 and dopamine levels [39]. 

To corroborate the neuroprotective capacity of *Enterococcus faecium* and agave inulin supplementation, we evaluated the size of the infarct, which was significantly smaller in the treated group. This tissue preservation may be due to the reduction in pro-inflammatory cytokines like TNF-α, one of the main inflammatory mediators that facilitates the infiltration of peripheral leukocytes by increasing permeability and inducing the rupture of the BBB, leading to apoptosis [26]. In the same way, the reduction in leukocyte infiltration could diminish the attack of free radicals. Therefore, a reduction in TNF-α levels contributes to a reduction in infarct volume, leading to greater neurological recovery and preserving neural functions in the hippocampus, such as memory. 

Similarly, symbiotic supplementation also enhanced spatial and working memory recovery. These data are very similar to the results obtained by the group of Lee et al., who performed a fecal transplant from young mice with a healthy intestinal microbiome to aged mice subjected to tMCAO. This study showed improvement in behavior and memory two weeks after the intervention. Furthermore, 11 days after the transplant, the microbiome of the mice with tMCAO was very similar to that of the donors [40]. 

After stroke, inflammation causes alterations in the microenvironment of the hypothalamus that directly affect the functioning of the hypothalamic neurons, mainly affecting memory. The process of neuroinflammation has been observed persisting into the chronic stages. Radenovic et al. reported the presence of activated microglial cells and astrocytes up to two years post-ischemia, particularly notable in the CA1 and CA3 regions of the hippocampus. These findings are associated with progressive neurodegeneration and suggest a potential link to the development of dementia [4]. Previous studies have shown that the symbiotic inulin and *E. faecium* can reduce inflammation in this area of the brain and induce the expression of neurotrophic factors [15]. The results of our present work are consistent with these findings. 

Furthermore, BDNF is capable of inducing neuroplasticity and is involved in long-term potentiation (LTP) processes during memory recall and learning [15]. These effects indicate the possible mechanisms that may be involved in cognitive recovery after an ischemic event, since BDNF is a key molecule for memory establishment. 

The findings of this study represent the first approach analyzing the effect of supplementation with *E. faecium* and agave inulin after cerebral ischemia. These promising results provide some bases to contemplate the potential impact of this therapeutic strategy on the chronic phase of ischemia [4]. In line with this, it will be necessary to plan the assessment of diverse parameters including both local and systemic inflammation and morphological alteration, among others. 

## 5. Conclusions

Supplementation with the symbiotic *E. faecium* and agave inulin can induce neuroprotection (infarct size reduction) in a model of cerebral ischemia. This could be the result of reducing the TNF-α concentrations and increasing BDNF expression. This neuroprotective effect promoted better neurological recovery. In the same way, symbiotic supplementation induced a significant recovery of spatial and working memory. Therefore, symbiotic supplementation could work as an adjuvant therapy to improve neurological recovery in stroke patients. Further research is needed to provide more evidence supporting the usefulness of this therapeutic strategy. 

## Data Availability

The data presented in this study are available on request from the corresponding author.
